# Increased Serum Angiopoietin-like Peptide 4 in Impaired Glucose Tolerance and Diabetes Subjects with or Without Hepatic Steatosis

**DOI:** 10.3390/jcm14217599

**Published:** 2025-10-26

**Authors:** Meng-Wei Lin, Chung-Hao Li, Hung-Tsung Wu, Chun-Te Lee, Huang-Pin Chen, Horng-Yih Ou, Hsin-Yu Kuo

**Affiliations:** 1 Department of Internal Medicine, National Cheng Kung University Hospital, College of Medicine, National Cheng Kung University, Tainan 704, Taiwan; n104893@mail.hosp.ncku.edu.tw (M.-W.L.); n048074@mail.hosp.ncku.edu.tw (C.-T.L.); n108108@mail.hosp.ncku.edu.tw (H.-P.C.); 2 School of Medicine, College of Medicine, China Medical University, Taichung 404, Taiwan; 070229@tool.caaumed.org.tw; 3 Department of Family Medicine, Taichung Municipal Geriatric Rehabilitation General Hospital-Managed by China Medical University, Taichung 406, Taiwan; 4 Department of Internal Medicine, School of Medicine, College of Medicine, National Cheng Kung University, Tainan 701, Taiwan; z11008014@ncku.edu.tw; 5 Tong-Yuan Diabetes Center, College of Medicine, National Cheng Kung University, Tainan 701, Taiwan

**Keywords:** angiopoietin-like peptide 4, diabetes, glycemic status, glucose intolerance, hepatic steatosis

## Abstract

**Objectives:** Although angiopoietin-like 4 (ANGPTL4) is highly associated with glucose hemostasis and lipid metabolism, the relationships between the serum ANGPTL4 level, glucose status and hepatic steatosis remain unclear. Therefore, this study aimed to quantify the independent effects of glucose intolerance and hepatic steatosis on circulating ANGPTL4 concentrations. **Methods:** A total of 348 age- and sex-matched participants with normal glucose tolerance (NGT), impaired fasting glucose (IFG), impaired glucose tolerance (IGT) and newly diagnosed diabetes (NDD) with or without hepatic steatosis were recruited for this cross-sectional study. Serum ANGPTL4 levels were measured, and multivariate linear regression analysis was used to evaluate the relationship between ANGPTL4, glycemic status and hepatic steatosis. **Results:** Compared with NGT, both IGT and NDD were associated with significantly higher serum ANGPTL4 concentrations, irrespective of hepatic steatosis status. Serum ANGPTL4 did not differ by the presence versus absence of hepatic steatosis. In multiple regression analysis, body mass index, homeostasis model assessment of insulin resistance, NGT vs. IGT, and NGT vs. NDD were independently associated with ANGPTL4 levels after adjustment for cardiovascular risk factors and adiponectin, whereas hepatic steatosis was not. **Conclusions:** Elevated serum ANGPTL4 concentrations were independently associated with prediabetes and diabetes, irrespective of hepatic steatosis.

## 1. Introduction

Hepatic steatosis has emerged as a major global health concern amid rising rates of obesity and unhealthy lifestyles. When it co-occurs with cardiometabolic risk factors such as obesity, hypertension, or hypertriglyceridemia, it is associated with increased risks of cardiovascular disease, liver cirrhosis, and hepatocellular carcinoma [1]. In Taiwan, a longitudinal cohort using ultrasonographic diagnosis reported relative risks of 3.51 for cirrhosis and 1.91 for hepatocellular carcinoma among individuals with hepatic steatosis compared with those without [2]. Insulin resistance plays an important role in the pathogenesis of hepatic steatosis. A meta-analysis of 156 studies estimated that 65.04% of patients with type 2 diabetes mellitus have hepatic steatosis [3]. Mechanistically, insulin resistance can disrupt endoplasmic reticulum–mitochondria communication [4,5], thereby promoting hepatic lipid accumulation [6].

Angiopoietin-like 4 (ANGPTL4) plays an important role in glucose homeostasis and lipid metabolism [7]. It is expressed across multiple tissues, including adipose tissue and the liver; its transcription is regulated by the lipid-sensing peroxisome proliferator–activated receptors (PPARs) α, β, and γ and is inducible by free fatty acids in vitro [8,9,10]. Functionally, ANGPTL4 is an endogenous inhibitor of lipoprotein lipase (LPL), the key enzyme mediating the clearance of triglyceride-rich plasma lipoproteins [8,11]. LPL, located on the luminal surface of the capillary endothelium, catalyzes the hydrolysis of endogenous very-low-density lipoprotein-triglyceride and exogenous chylomicron-triglyceride to glycerol and free fatty acids [12,13]. Therefore, ANGPTL4 influences circulating free fatty acids by modulating LPL activity. Excess free fatty acids will be taken up by peripheral tissues, including the liver, where they undergo β-oxidation for energy or are re-esterified and stored as triglycerides, thereby contributing to hepatic lipid accumulation [14]. In animal models, genetic inactivation of ANGPTL4 improves insulin sensitivity and glucose homeostasis even under high-fat-diet feeding [15,16]. In human cohorts, higher circulating ANGPTL4 concentrations have been observed in individuals with metabolic syndrome or type 2 diabetes compared with healthy controls [17,18], whereas patients with hepatic steatosis may exhibit lower plasma ANGPTL4 levels, potentially reflecting diminished LPL inhibition and increased hepatic fatty acid influx [19].

There is a bidirectional relationship between hepatic steatosis and type 2 diabetes mellitus that is confirmed by epidemiological data, clinical picture, diagnosis and pathomechanisms [20]. However, the complex causal link between serum ANGPTL4 concentrations, hepatic steatosis and type 2 diabetes mellitus remain unclear. Accordingly, this study aims to quantify the independent effects of glycemic status and hepatic steatosis on circulating ANGPTL4 concentrations and to assess their potential interaction.

## 2. Materials and Methods

### 2.1. Study Population

The study was approved from the Institutional Review Board of National Cheng Kung University Hospital, Tainan, Taiwan (B-ER-102-418, A-ER-105-139 and B-ER-111-096), and conducted in compliance with the International Conference on Harmonisation guidelines for Good Clinical Practice and adhered to the principles outlined in the Declaration of Helsinki.

This case–controlled study enrolled participants at National Cheng Kung University Hospital between Dec 2009 and July 2019 in a health check-up center and outpatient clinic. All participants first underwent standard fasting blood examinations, followed by a standard 75 g oral glucose tolerance test (OGTT) as part of the standardized health check-up protocol. In accordance with diagnostic guidelines, individuals with newly diagnosed diabetes based on a fasting glucose level more than 7.0 mmol/L (126 mg/dL) did not undergo OGTT. From this cohort, we subsequently matched across groups by age and sex. Each diabetic participant was matched with the first available individual of the same sex and either identical or closest age (±1 year) from the remaining eligible population. Furthermore, participants were enrolled and stratified into eight categories based on glycemic status and the presence or absence of hepatic steatosis: normal glucose tolerance (NGT), impaired fasting glucose (IFG), impaired glucose tolerance (IGT), newly diagnosed diabetes (NDD), NGT with hepatic steatosis, IFG with hepatic steatosis, IGT with hepatic steatosis, and NDD with hepatic steatosis. Classification was performed according to the diagnostic criteria of the American Diabetes Association (ADA) using the results of a OGTT [21]. Specifically, individuals were classified as NGT if their fasting plasma glucose (FPG) was <5.6 mmol/L (100 mg/dL) and their 2 h postload glucose was <7.8 mmol/L (140 mg/dL), with no prior history of diabetes. IFG was defined as an FPG level between 5.6 and 6.9 mmol/L (100–125 mg/dL) and a 2 h postload glucose < 7.8 mmol/L (140 mg/dL). IGT was characterized by an FPG < 5.6 mmol/L (100 mg/dL) with a 2 h postload glucose ranging from 7.8 to 11.1 mmol/L (140–199 mg/dL). All participants were restricted to isolated IFG or isolated IGT; individuals with both FPG 5.6 to 6.9 mmol/L and 2 h post-load glucose 7.8 to 11.1 mmol/L were excluded. NDD was diagnosed when FPG was ≥7.0 mmol/L (126 mg/dL) or 2 h postload glucose was ≥11.1 mmol/L (200 mg/dL). Hepatic steatosis was diagnosed by ultrasound-based techniques [22].

We excluded subjects meeting any of the following criteria: (1) alcohol intake ≥ 20 g/day within the preceding year; (2) serum aspartate aminotransferase (AST) or alanine aminotransferase (ALT) levels higher than two times the normal limit; (3) positive hepatitis B surface antigen, hepatitis C virus antibody, or other established causes of liver disease; (4) evidence of acute or chronic inflammatory conditions, defined as a leukocyte count over 10,000/mm^3^ or clinical signs of infection; or (5) any other major diseases, including generalized inflammation or advanced malignant diseases.

### 2.2. Data Review

Data on demographic characteristics, medical history, and lifestyle habits were obtained through standardized questionnaires administered by trained study nurses. Baseline variables included age, sex, comorbidities, body mass index (BMI; calculated as weight in kilograms divided by height in m^2^), alcohol consumption, smoking status, and habitual exercise. Alcohol consumption was defined as the intake of alcoholic beverages less than 20 g per day. Smoking was defined as the consumption of at least one pack (20 cigarettes) per month over the preceding six months. Habitual exercise was defined as engaging in vigorous physical activity sufficient to induce sweating more than three times per week. Hypertension was defined as persistently elevated blood pressure ≥ 130/80 mmHg [23] and obesity was defined as body mass index ≥ 27.0 [24].

Following a 12 h overnight fast, all participants underwent blood sampling in the morning at a standardized time to ensure sample stability. After centrifugation, serum was divided into several aliquots and stored at −80 °C until analysis to prevent repeated freeze–thaw cycles. The conditions of storage were identical for all participants. For the present analysis, only previously unthawed serum aliquots were retrieved and analyzed after age- and sex-matching of participants. Blood glucose was quantified using the hexokinase method (Roche Diagnostic GmbH, Mannheim, Germany). Serum ANGPTL4 concentrations were determined using a commercially available enzyme-linked immunosorbent assay (ELISA) kit (intra-assay CV < 8.8%, inter-assay CV < 11.2%; R&D Systems, Minneapolis, MN, USA). The assay is designed to detect total ANGPTL4 concentrations in human serum and does not differentiate between the full-length protein and its cleaved fragments. Serum adiponectin (intra-assay CV < 2.5%, inter-assay CV < 6.5%; AssayPro, St. Charles, MO, USA) and high-sensitivity C-reactive protein (intra-assay CV < 2.9%, inter-assay CV < 4.7%; Immunology Consultants Laboratory, Newberg, OR, USA) were measured using commercially available sandwich enzyme-linked immunosorbent assay kits. Insulin resistance was estimated using the homeostasis model assessment of insulin resistance (HOMA-IR) index, calculated as [fasting insulin (pmol/L) × FPG (mmol/L)]/135 [25]. Serum lipid profiles were determined with an automated analyzer (Hitachi 747E; Hitachi, Tokyo, Japan). These variables were subsequently analyzed to identify factors independently associated with serum ANGPTL4 levels and to examine the relationship between serum ANGPTL4 levels and glycemic status with or without hepatic steatosis.

### 2.3. Statistical Analysis

Data are presented as the mean ± standard deviation (SD) for normally distributed variables and or median, upper and lower quartiles for non-normally distributed variables, or number (percentage) depending on the types of variables. Differences in baseline characteristics were analyzed using the chi-square test or Student’s *t*-test. The continuous variables among groups were compared using one-way analysis of variance (ANOVA) with Bonferroni-corrected two-tailed Student’s *t*-test as post hoc tests. To further determine the independent factors associated with serum ANGPTL4 levels, multivariate linear regression analysis was performed. Covariates were selected based on their established relevance to ANGPTL4 regulation, encompassing parameters that reflect glucose metabolism, inflammatory status, and other clinical characteristics related to hepatic steatosis and general characteristics. In Model 1, we included general parameters such as sex and age, together with continuous variables representing glucose metabolism, inflammatory status, liver function, cardiovascular function, and renal function, to perform a multivariate linear regression analysis. In Model 2, the same general parameters were retained, while the continuous variables were converted into categorical variables to conduct a separate multivariate linear regression analysis, allowing assessment of the association between serum ANGPTL4 levels and categorized clinical parameters. *p*-values less than 0.05 were considered statistically significant. Finally, to assess the predictive performance of serum ANGPTL4, we generated receiver-operating characteristic (ROC) curves and calculated the area under the curve (AUC) with 95% confidence intervals. The Youden index was applied to identify the optimal cut-off value. All data analyses were performed using SPSS version 20.0 statistical software (IBM Corp., Armonk, NY, USA).

## 3. Results

### 3.1. Baseline Characteristics

A total of 348 age- and sex-matched participants with NGT (*n* = 45), IFG (*n* = 38), IGT (*n* = 42), NDD (*n* = 46), NGT + hepatic steatosis (*n* = 45), IFG + hepatic steatosis (*n* = 43), IGT + hepatic steatosis (*n* = 47), NDD + hepatic steatosis (*n* = 42) were included in the final analysis. The majority were male (61.2%), with a mean age of 61.0 ± 11.1 years and a mean BMI of 25.2 ± 3.4 kg/m^2^. In addition, 177 participants (50.9%) had hepatic steatosis. The prevalence of obesity was significantly higher in participants with hepatic steatosis than in those without (*p* < 0.001).

At baseline, significant differences were observed among different groups in terms of BMI (*p* < 0.001), HbA1c (*p* < 0.001), ANGPTL4 (*p* < 0.001), HOMA-IR (*p* < 0.001), ALT (*p* < 0.001), AST (*p* < 0.001), high-sensitivity C-reactive protein (*p* = 0.002), triacylglycerol (*p* = 0.007), HDL (*p* < 0.001), and adiponectin (*p* < 0.001). In contrast, no statistically significant differences were found across the eight groups with respect to gender (*p* = 0.998), age (*p* = 0.681), hypertension (*p* = 0.423), creatinine (*p* = 0.126), total cholesterol (*p* = 0.981), LDL (*p* = 0.981), smoking status (*p* = 0.706), habitual exercise (*p* = 0.267), or alcohol use (*p* = 0.239). The detailed baseline characteristics of the study population are presented in Table 1.

### 3.2. Participants with IGT and NDD Had Significantly Higher Serum ANGPTL4 Concentrations than Those with NGT, Irrespective of Hepatic Steatosis Status

**Figure 1:**
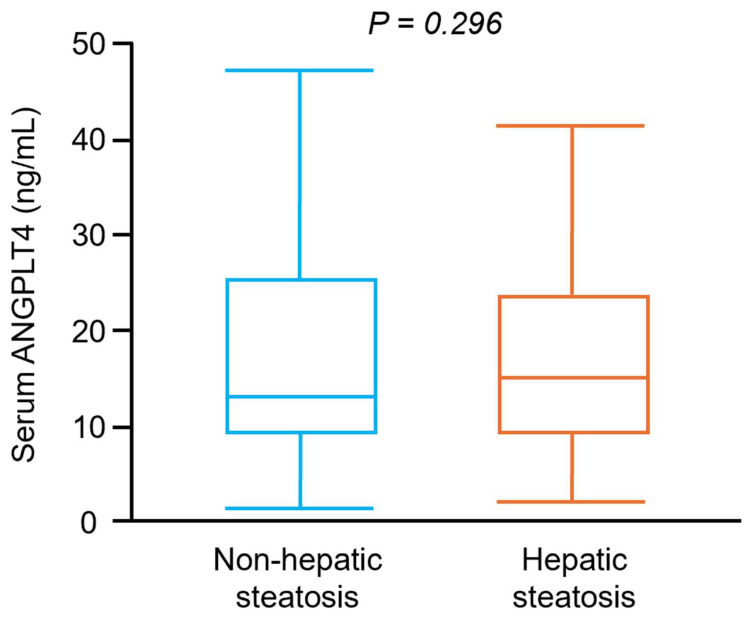
Serum ANGPTL4 concentrations in subjects with and without hepatic steatosis. Box and whisker plot of serum ANGPTL4 concentrations in participants without hepatic steatosis (*n* = 171) and with hepatic steatosis (*n* = 177).

**Figure 2:**
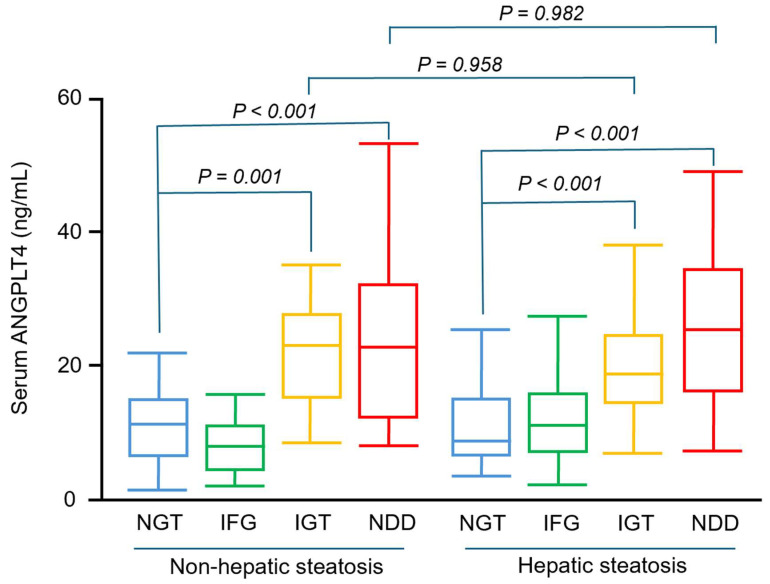
Plasma ANGPTL4 concentrations in prediabetes or diabetes participants with or without hepatic steatosis. Box and whisker plot of serum ANGPTL4 concentrations in participants with NGT (*n* = 45), IFG (*n* = 38), IGT (*n* = 42), NDD (*n* = 46), NGT combine hepatic steatosis (*n* = 45), IFG combine hepatic steatosis (*n* = 43), IFG combine hepatic steatosis (*n* = 47), NDD combine hepatic steatosis (*n* = 42). The line inside the box represents the median of the distribution, the box top and bottom values are defined by the 25th and 75th percentiles, and the whiskers are the minimum and maximum values. NGT, normal glucose tolerance; IFG, impaired fasting glucose; IGT, impaired glucose tolerance; NDD, newly diagnosed diabetes; HS, hepatic steatosis.

Overall, serum ANGPTL4 concentrations did not differ significantly between participants with and without hepatic steatosis (Figure 1). As shown in Figure 2, serum ANGPTL4 concentrations were 11.2 ± 5.4, 11.7 ± 16.7, 23.8 ± 11.9, 29.6 ± 26.9, 10.9 ± 5.8, 14.3 ± 14.5, 20.4 ± 7.9, 25.0 ± 10.9 ng/mL in subjects with NGT, IFG, IGT, NDD, NGT + hepatic steatosis, IFG + hepatic steatosis, IGT + hepatic steatosis, NDD + hepatic steatosis, respectively.

In subgroup analyses stratified by hepatic steatosis, participants without steatosis exhibited higher serum ANGPTL4 concentrations in the IGT (*p* < 0.001) and NDD (*p* = 0.001) groups compared with NGT; similar associations were observed among participants with hepatic steatosis (both *p* < 0.001 for IGT and NDD compared with NGT). Within each steatosis stratum, ANGPTL4 did not differ significantly between IGT and NDD (*p* = 0.995 without steatosis; *p* = 0.535 with steatosis; Figure 2). In ROC analyses, serum ANGPTL4 discriminated glucose intolerance with an AUC of 0.838 (95% CI, 0.797–0.879). The Youden index identified an optimal cut-off of 13.925, yielding 75.7% sensitivity and 74.9% specificity.

### 3.3. Prediabetes and Diabetes Were Independently Associated with Higher Serum ANGPTL4 Concentrations, Whereas Hepatic Steatosis Was Not

To determine the factors independently associated with serum ANGPTL4 concentrations, we conducted multivariable linear regression. As shown in Table 2, serum ANGPTL4 was positively associated with HOMA-IR (β = 1.57; 95% CI, 0.10–3.03; *p* = 0.036) and BMI (β = 0.89; 95% CI, 0.32–1.46; *p* = 0.002) after adjustment for the covariates specified in Model 1. We specified Model 2 using NGT as the reference group and adjusting for hepatic steatosis, hypertension, obesity, chronic kidney disease, smoking status, habitual exercise, and alcohol use. In Model 2, both IGT (β = 11.76; 95% CI, 7.55–15.98; *p* < 0.001) and NDD (β = 16.49; 95% CI, 12.24–20.73; *p* < 0.001) were independently associated with higher ANGPTL4 concentrations. In contrast, the presence of hepatic steatosis was not significantly associated with ANGPTL4 concentrations.

## 4. Discussion

To the best of our knowledge, this is the first study to explore the independent effects of hepatic steatosis and glycemic status on serum ANGPTL4 concentrations. We found that IGT and NDD were independently associated with higher serum ANGPTL4 concentrations, irrespective of hepatic steatosis.

Prior studies have linked circulating ANGPTL4 to glucose metabolism. In ANGPTL4-deficient animals, genetic depletion improves glucose homeostasis and lowers atherosclerotic plaque burden [16,26,27,28]. In humans, loss-of-function variation in ANGPTL4, particularly p.E40K, is associated with reduced risk of type 2 diabetes and improved insulin sensitivity [15], whereas higher circulating ANGPTL4 has been reported among individuals with type 2 diabetes and metabolic syndrome compared with healthy controls [11,29,30,31]. Consistent with these observations, we found that participants with prediabetes or type 2 diabetes had higher serum ANGPTL4 concentrations than healthy controls, irrespective of hepatic steatosis status. One possible mechanism is that glucose intolerance is often accompanied by elevated plasma free fatty acids and ANGPTL4 expression in multiple tissues, including liver and adipose tissue, thought to be primarily regulated by peroxisome proliferator-activated receptors (PPARs). Consistent with this, fatty acids upregulate ANGPTL4 in vitro across diverse cell types [7,10,32]. Moreover, in vivo manipulation of plasma free fatty acid levels produces concordant changes in circulating ANGPTL4, with a positive correlation between free fatty acid and ANGPTL4 changes [33,34]. Taken together, our observations align with prior mechanistic work, suggesting that glycemic status is the principal correlate of ANGPTL4.

ANGPTL4 plays a central role in lipid metabolism by inhibiting lipoprotein lipase (LPL), thereby promoting an increase in circulating free fatty acid levels [11,35,36]. The released FFAs are taken up by multiple organs, including the liver, where they undergo β-oxidation or are re-esterified and stored as triglycerides, contributing to hepatic lipid accumulation. One study reported lower ANGPTL4 concentrations in adults with hepatic steatosis compared with healthy controls, positing that reduced ANGPTL4 leads to disinhibition of LPL, enhanced hydrolysis of circulating triglycerides, and increased free fatty acid flux to the liver [19]. In our cohort, however, when participants were grouped by the presence or absence of hepatic steatosis, serum ANGPTL4 concentrations, although numerically lower in steatosis, did not differ significantly between groups. Several prior reports similarly found no significant difference between circulating ANGPTL4 and hepatic steatosis [37]. These observations suggest that additional factors, such as glucose intolerance, may be more salient determinants of ANGPTL4 levels. Accordingly, we further classified participants by glycemic status with and without hepatic steatosis. We observed significantly higher serum ANGPTL4 concentrations in individuals with prediabetes and diabetes, irrespective of steatosis status. Moreover, among those with glucose intolerance, ANGPTL4 concentrations were comparable between participants with and without hepatic steatosis. Collectively, these findings implicate glycemic status, rather than steatosis per se, better explains interindividual variation in circulating ANGPTL4.

To further evaluate the relationships among glycemic status, hepatic steatosis, and serum ANGPTL4 concentrations, we performed multivariable linear regression. Prediabetes and diabetes were independently associated with higher serum ANGPTL4, whereas the presence of hepatic steatosis was not. HOMA-IR and BMI also emerged as independent factors, consistent with prior studies [37,38,39]. Our findings may give future a foundation for future research. Insulin and ANGPTL4 are key, opposing regulators of LPL activity, insulin enhances LPL, whereas ANGPTL4 inhibits it, and LPL activity is closely linked to circulating free fatty acid levels and the development of hepatic steatosis. Although ANGPTL4 is known to regulate LPL, it remains unclear whether alterations in LPL activity driven by other factors (e.g., insulin, circulating free fatty acids) will negatively feedback to modulate ANGPTL4 expression and requires further investigation.

In addition to ANGPTL4, other angiopoietin-like proteins, notably ANGPTL3 and ANGPTL8, are also important to lipid regulation. Emerging evidence supports a coordinated ANGPTL3–4–8 axis in which ANGPTL3 and ANGPTL8 act systemically, while ANGPTL8 also modulates ANGPTL4 locally to fine-tune LPL activity [40]. In the fed state, hepatically secreted ANGPTL3–ANGPTL8 complexes inhibit LPL with substantially greater potency than ANGPTL3 alone, explaining why loss of either component lowers plasma triglycerides and why the complex is considered the physiologically relevant circulating LPL inhibitor [40,41]. Within adipose tissue, ANGPTL8 physically interacts with ANGPTL4 to form ANGPTL4/8 complexes that affect ANGPTL4-mediated LPL inhibition, thereby preserving adipose LPL activity after feeding [40]. Extending beyond lipid handling, adipocyte-specific ANGPTL8 deletion in diet-challenged mice improves whole-body glucose homeostasis, trends toward enhanced insulin sensitivity, and increases insulin-stimulated glucose uptake in adipose tissue [42]. Clinical observations in diabetes further show that high circulating ANGPTL8 associates with higher triglyceride levels and insulin resistance, and that concurrent high ANGPTL3 and ANGPTL8 identifies elevated TG and LDL [43,44]. This highlighting the clinical relevance of ANGPTL3/8–ANGPTL4/8 crosstalk to both lipid metabolism and glucose homeostasis.

A comparison can also be made with other biomarkers of glucose intolerance. Hepatocyte-derived fibrinogen-related protein-1 (HFREP1) has been proposed as a hepatokine linking hepatic metabolic stress to systemic glucose homeostasis. Elevated circulating HFREP1 concentrations have been reported in individuals with impaired glucose tolerance and newly diagnosed diabetes, with positive associations with indices of insulin resistance, suggesting potential utility as a biomarker for glucose intolerance [45,46,47]. However, HFREP1 expression is strongly liver-dependent; patients with viral hepatitis, hepatic steatosis, or drug-induced liver injury frequently exhibit increased hepatic HFREP1 as part of generalized acute-phase or regenerative responses, which may confound its interpretation in metabolic screening [48,49]. In contrast, in our cohort the presence of hepatic steatosis did not modify the elevation of serum ANGPTL4 observed in participants with prediabetes and diabetes. Fetuin-A has likewise been linked to metabolic dysregulation and glucose intolerance; prior studies show that Fetuin-A concentrations are independently associated with glucose intolerance and correlate positively with triglycerides, BMI, and HOMA-IR [50]. Mechanistically, Fetuin-A inhibits insulin-receptor tyrosine kinase activity and amplifies lipid-induced inflammation and insulin resistance [51]. Yet Fetuin-A levels are strongly influenced by hepatic fat accumulation, obesity, and systemic inflammation, complicating clinical interpretation and suggesting that Fetuin-A may function more as a marker of metabolic dysfunction than a direct causal mediator [50,52,53]. Taken together, while HFREP1 and Fetuin-A provide informative hepatic and systemic signals of metabolic stress, our data indicates that ANGPTL4 is elevated in glucose intolerance independently of hepatic steatosis.

Worldwide, an estimated 240 million individuals have undiagnosed diabetes, and nearly half of all adults with diabetes are unaware of their condition [47]. Many patients also have suboptimal control of glycemia and associated cardiovascular risk factors [54,55]. Although the reasons are multifactorial [56,57], delayed recognition along the natural history of dysglycemia contributes substantially [58]. Accordingly, early identification at the prediabetes stage is critical, enabling lifestyle modification or pharmacotherapy to maintain near-normal glycemia, delay or prevent diabetes onset, and reduce cardiovascular morbidity and mortality [59,60]. The OGTT is considered the gold standard for diagnosing prediabetes and diabetes, but its clinical utility is limited by fasting requirements and procedural inconvenience [61]. Hemoglobin A1c is useful, yet limitations persist, notably intra-subject variability even with National Glycohemoglobin Standardization Program (NGSP)-certified methods in obese youth [62] and reduced reliability in conditions with altered red-cell turnover such as hemoglobinopathies, anemia, pregnancy, recent blood loss or transfusion, hemolysis, and erythropoietin use [63]. In our study, serum ANGPTL4 concentrations were significantly higher in individuals with glucose intolerance. Multivariable regression further showed that both prediabetes and diabetes were independently associated with higher ANGPTL4 levels. These findings demonstrate an association between serum ANGPTL4 and glucose intolerance. Prospective, longitudinal studies with mechanistic evaluation are warranted to determine whether this relationship has predictive or diagnostic implications. In routine health check-up settings, measurement of serum ANGPTL4 may, pending external validation, serve as a complementary screening aid to identify individuals with possible glucose intolerance, given its practical convenience in clinical workflows. With respect to cost-effectiveness, a formal health-economic analysis was not prespecified and lies beyond the scope of this cross-sectional study. Because test costs, laboratory workflows (including sample processing and cold-chain storage), reimbursement, and downstream utilization vary by setting and platform, data were not collected. Accordingly, the economic value of incorporating ANGPTL4 into diagnostic pathways remains to be established. Future research should include prospective outcome studies followed by decision-analytic modeling to assess whether adding ANGPTL4 yields incremental clinical value.

This study has some limitations. First, hepatic steatosis was diagnosed by ultrasonography rather than histology. Although liver biopsy remains the diagnostic gold standard, it is impractical for routine use; by contrast, ultrasound is an established, noninvasive screening tool with acceptable sensitivity and specificity [24], but it is operator-dependent and less sensitive to mild fat infiltration. To reduce interobserver variability, all examinations were performed by a single experienced radiologist. Second, we did not perform subgroup analyses by steatosis severity because ultrasonography does not permit precise quantification of hepatic fat burden. Third, the cross-sectional design precludes causal inference regarding the relationships among circulating ANGPTL4, glycemic status, and hepatic steatosis. Despite these constraints, the study has several strengths: to our knowledge, it is the first to assess the independent and joint associations of ANGPTL4 with glycemic status and hepatic steatosis, and it enrolled a larger sample than prior investigations.

Taken together, we observed higher circulating ANGPTL4 concentrations in individuals with prediabetes and diabetes, irrespective of hepatic steatosis. In multivariable models, glucose intolerance was independently associated with serum ANGPTL4 concentrations, suggesting a potential physiological link between ANGPTL4 and glucose metabolism that warrants further investigation.

## Figures and Tables

**Table 1:** Clinical characteristics of the study participants in each group.

Characteristics	NGT (*n* = 45)	IFG (*n* = 38)	IGT (*n* = 42)	NDD (*n* = 46)	NGT + Hepatic Steatosis (*n* = 45)	IFG + Hepatic Steatosis (*n* = 43)	IGT + Hepatic Steatosis (*n* = 47)	NDD + Hepatic Steatosis (*n* = 42)	*p*
Male, %	64.4	63.2	57.1	58.7	60.0	62.8	61.7	61.9	0.998
Age, year	61.3 ± 11.7	61.0 ± 11.9	62.7 ± 11.5	61.7 ± 11.3	61.4 ± 10.4	60.8 ± 10.2	61.0 ± 11.0	61.2 ± 10.7	0.681
BMI, kg/m^2^	22.9 ± 2.8	23.9 ± 2.2	23.7 ± 3.4	23.5 ± 3.1	26.1 ± 3.2	26.7 ± 2.8	26.8 ± 2.8	27.5 ± 2.9	<0.001
HTN, %	28.9	18/4	21.4	21.7	13.3	30.2	29.8	31.0	0.423
CKD, %	3.3	5.0	1.9	4.8	5.3	4.2	4.1	5.1	0.235
Obesity, %	4.5	11.4	14.3	15.2	42.2	36.6	46.8	45.2	<0.001
HbA1c, %	5.5 ± 0.4	5.9 ± 0.4	5.9 ± 0.3	7.2 ± 2.1	5.5 ± 0.3	5.9 ± 0.4	5.9 ± 0.4	7.6 ± 1.7	<0.001
HOMA-IR	0.6 ± 0.6	0.8 ± 0.6	1.3 ± 0.5	1.5 ± 0.9	0.7 ± 0.5	1.2 ± 0.6	1.3 ± 0.9	2.9 ± 2.4	<0.001
ALT, U/L	24.0 ± 11.1	24.8 ± 19.9	20.7 ± 6.9	24.9 ± 9.3	30.1 ± 11.4	32.2 ± 19.9	36.2 ± 20.4	43.3 ± 29.9	<0.001
AST, U/L	25.6 ± 6.8	26.0 ± 8.7	23.4 ± 4.6	24.9 ± 6.9	27.7 ± 7.7	26.6 ± 7.9	30.1 ± 9.6	34.3 ± 16.6	<0.001
SBP, mmHg	123.6 ± 17.3	126.6 ± 17.3	128.5 ± 17.7	126.5 ± 16.6	124.8 ± 14.8	131.0 ± 16.1	132.3 ± 13.5	130.0 ± 12.6	0.247
Creatinine, μmol/L	76.5 ± 15.2	77.3 ± 17.1	78.8 ± 16.3	75.3 ± 21.1	77.1 ± 14.5	77.1 ± 13.7	76.3 ± 18.5	78.5 ± 19.2	0.126
Albumin, µmol/L	654.7 ± 36.5	669.2 ± 32.9	650.0 ± 38.9	656.2 ± 49.7	653.4 ± 35.5	671.6 ± 39.9	676.2 ± 35.4	667.6 ± 41.9	0.226
hs-CRP, nmol/L	27.1 ± 40.7	26.9 ± 16.7	28.0 ± 46.4	62.5 ± 93.2	26.3 ± 81.6	33.1 ± 41.5	33.2 ± 31.8	62.9 ± 77.4	0.002
Total cholesterol, mmol/L	5.4 ± 1.1	5.4 ± 1.0	5.3 ± 1.0	5.4 ± 1.2	5.5 ± 1.1	5.4 ± 1.0	5.5 ± 1.0	5.3 ± 1.0	0.981
TG, mmol/L ^a^	1.1 ± 0.4	1.3 ± 0.9	1.3 ± 0.7	1.6 ± 1.3	1.8 ± 0.8	1.7 ± 1.3	1.9 ± 1.0	2.1 ± 1.8	0.007
HDL-cholesterol, mmol/L	1.5 ± 0.4	1.5 ± 0.4	1.4 ± 0.4	1.3 ± 0.3	1.2 ± 0.2	1.2 ± 0.4	1.2 ± 0.3	1.2 ± 0.3	<0.001
LDL-cholesterol, mmol/L	3.3 ± 1.0	3.3 ± 1.0	3.3 ± 0.9	3.4 ± 1.0	3.4 ± 1.0	3.4 ± 0.9	3.4 ± 0.8	3.3 ± 0.8	0.894
Adiponectin, μmol/dL	12.9 ± 12.8	14.4 ± 9.3	10.5 ± 4.9	10.1 ± 5.6	10.0 ± 9.5	10.2 ± 3.4	8.9 ± 4.8	7.4 ± 3.7	<0.001
Smoking, %	4.4	5.3	7.1	8.7	8.9	7.0	12.8	2.4	0.706
Habitual exercise, %	4.4	5.3	9.5	2.2	2.2	0.0	4.3	0.0	0.267
Alcohol use, %	2.2	0.0	2.4	2.2	0.0	0.0	6.4	0.0	0.239

^a^ Kruskal–Wallis test; ALT, alanine aminotransferase; AST, aspartate aminotransferase; BMI, body-mass index; CKD, chronic kidney disease; HTN, hypertension; HDL, high-density lipoprotein; HOMA-IR, homeostasis model assessment of insulin resistance; hs-CRP, high-sensitivity C-reactive protein; LDL, low-density lipoprotein; SBP, systolic blood pressure; TG, triglyceride.

**Table 2:** Regression analysis between plasma ANGPTL4 concentrations and clinical variables.

Variable	Model 1		Model 2	
	β (95% CI)	*p*	β (95% CI)	*p*
Sex	0.56 (−4.30, 5.42)	0.821	3.15 (−0.12, 6.42)	0.059
Age	−0.03 (−0.20, 0.14)	0.734	0.02 (−0.15, 0.19)	0.962
HOMA-IR	1.57 (0.10, 3.03)	0.036		
Body-mass index	0.89 (0.32, 1.46)	0.002		
Adiponectin	−0.08 (−0.32, 0.16)	0.498		
hs-CRP	0.02 (−0.01, 0.04)	0.247	0.01 (−0.02, 0.04)	0.477
Triacylglycerol	−0.89 (−2.53, 0.76)	0.292		
HDL-cholesterol	−2.66 (−8.26, 2.94)	0.351		
SBP	0.01 (−0.11, 0.12)	0.934		
ALT	0.04 (−0.06, 0.13)	0.434		
Albumin	−7.32 (−13.986, −0.644)	0.032		
Creatinine	0.11 (−0.02, 0.25)	0.097		
IFG vs. NGT			2.34 (−2.07, 6.75)	0.297
IGT vs. NGT			11.76 (7.55, 15.98)	<0.001
NDD vs. NGT			16.49 (12.24, 20.73)	<0.001
Hepatic steatosis			−0.86 (−4.12, 2.40)	0.605
Obesity			−2.91 (−6.59, 0.77)	0.121
HTN			−0.02 (−3.64, 3.61)	0.994
CKD			1.32 (−1.94, 4.57)	0.427
Smoking			1.69 (−4.84, 8.23)	0.510
Habitual exercise			−5.03 (−14.51, 4.45)	0.297
Alcohol use			−3.98 (−16.86, 8.90)	0.544

NGT, normal glucose tolerance; IFG, impaired fasting glucose; IGT, impaired glucose tolerance; NDD, newly diagnosed diabetes; ALT, alanine aminotransferase; CKD, chronic kidney disease; HTN, hypertension; HDL, high-density lipoprotein; HOMA-IR, homeostasis model assessment of insulin resistance; SBP, systolic blood pressure.

## Data Availability

All data generated in this study are available within this article or can be obtained from the corresponding author upon request.
